# Mild Type 2 Diabetes Mellitus Reduces the Susceptibility of the Heart to Ischemia/Reperfusion Injury: Identification of Underlying Gene Expression Changes

**DOI:** 10.1155/2015/396414

**Published:** 2015-07-01

**Authors:** Sevil Korkmaz-Icöz, Alice Lehner, Shiliang Li, Adrian Vater, Tamás Radovits, Péter Hegedűs, Mihály Ruppert, Paige Brlecic, Markus Zorn, Matthias Karck, Gábor Szabó

**Affiliations:** ^1^Department of Cardiac Surgery, University of Heidelberg, 69120 Heidelberg, Germany; ^2^Heart and Vascular Center, Semmelweis University, 1122 Budapest, Hungary; ^3^Department of Internal Medicine I, University of Heidelberg, 69120 Heidelberg, Germany

## Abstract

Despite clinical studies indicating that diabetic hearts are more sensitive to ischemia/reperfusion injury, experimental data is contradictory. Although mild diabetes prior to ischemia/reperfusion may induce a myocardial adaptation, further research is still needed. Nondiabetic Wistar (W) and type 2 diabetic Goto-Kakizaki (GK) rats (16-week-old) underwent 45 min occlusion of the left anterior descending coronary artery and 24 h reperfusion. The plasma glucose level was significantly higher in diabetic rats compared to the nondiabetics. Diabetes mellitus was associated with ventricular hypertrophy and increased interstitial fibrosis. Inducing myocardial infarction increased the glucose levels in diabetic compared to nondiabetic rats. Furthermore, the infarct size was smaller in GK rats than in the control group. Systolic and diastolic functions were impaired in W + MI and did not reach statistical significance in GK + MI animals compared to the corresponding controls. Among the 125 genes surveyed, 35 genes showed a significant change in expression in GK + MI compared to W + MI rats. Short-term diabetes promotes compensatory mechanisms that may provide cardioprotection against ischemia/reperfusion injury, at least in part, by increased antioxidants and the upregulation of the prosurvival PI3K/Akt pathway, by the downregulation of apoptotic genes, proinflammatory cytokine TNF-*α*, profibrogenic TGF-*β*, and hypertrophic marker *α*-actin-1.

## 1. Introduction

Diabetes mellitus (DM) is a common, chronic, metabolic syndrome characterized by hyperglycemia, resulting from defects in insulin secretion, insulin effects, or both. Type 2 diabetes accounts for more than 90% of all cases and is often associated with hyperinsulinaemia, hyperglycaemia, and dyslipidaemia. DM can affect the cardiac structure and function in the absence of hypertension, coronary artery disease, or hyperlipidemia, a phenomenon known as diabetic cardiomyopathy. Diabetic cardiomyopathy is characterized by ventricular dilatation, myocyte hypertrophy, prominent interstitial fibrosis, and decreased or sustained systolic function [1] in the presence of a diastolic dysfunction [2]. However, DM not only affects the myocardium, but also leads to vascular complications. Macrovascular complications of DM are responsible for the high incidence of vascular diseases, such as myocardial infarction, which is caused by an imbalance between the coronary blood flow and the metabolic demand of the myocardium. Clinical studies have shown that patients with DM are at a greater risk for myocardial infarction and that, after an infarction, diabetes increases the risk for the development of left-ventricular dysfunction and heart failure when compared with nondiabetic individuals [3, 4]. Additionally, morbidity and mortality rates after myocardial infarction are significantly higher in diabetic patients than in nondiabetic patients [5]. Myocardial infarct size is a primary determinant of the prognosis in patients with acute myocardial infarction. Although clinical studies showed that DM increased the susceptibility of the myocardium to ischemia/reperfusion injury [6, 7], infarct size studies in diabetic animal models showed quite contradictory results, especially at the early stage of the disease. It appears that the susceptibility of the postischemic myocardial injury of the diabetic heart is dependent on the duration of ischemia, the duration and the stage of the diabetes, and/or the metabolic profile. Evidence suggests that the ischemic hearts from the mildly diabetic animals are resistant to ischemia [8], and the severely diabetic hearts are more prone to ischemic injury [9]. Several mechanisms have been proposed to explain the cardioprotective property of a high glucose exposure, by an increase in survival pathways, such as antiapoptotic factor Bcl-2, and prosurvival factors such as protein kinase B (Akt), the inactivation of the proapoptotic factor bad, inappropriate expression of proangiogenic vascular endothelial growth factor (VEGF), hypoxia inducible factor 1*α*, and protein kinase C-*ε* [10].

The Goto-Kakizaki (GK) rat, a model of type 2 diabetes, was developed from a stock of Wistar rats, by selective breeding over many generations of rats with the highest blood glucose levels during an oral glucose tolerance test [11]. GK rats develop mild hyperglycemia and hyperinsulinemia and are not associated with the development of obesity, hypertension, or hyperlipidemia. The spontaneous diabetic GK rat thus offers a convenient model for studying the pathogenesis of diabetic cardiomyopathy, independent from the effect of hyperlipidemia, obesity, or hypertension.

Taking this into consideration, and in order to provide further insight into the understanding of the myocardial cellular/molecular events associated with short-term diabetes-induced cardioprotection, we investigated the effects of a myocardial ischemia/reperfusion injury on metabolic and functional changes and on cardiac gene expression patterns in GK rats. To that end, we evaluated* in vivo* the left-ventricular mechanical function with a Millar pressure-volume conductance catheter system, assessed biochemical and histopathological changes, and identified changes in myocardial gene expression relevant to diabetes, apoptosis, oxidative stress, PI3K/Akt signalling, inflammation, cardiac fibrosis, and hypertrophy using an RT^2^ Profiler PCR Arrays system.

## 2. Materials and Methods

### 2.1. Animals

Spontaneously diabetic male GK rats (Taconic, Ry, Denmark) and age-matched nondiabetic male Wistar (W) control rats (Charles River, Sulzfeld, Germany) were purchased at 4–9 weeks of age and studied at the age of 16 weeks. The animals were housed at a constant room temperature (22 ± 2°C) and 12 h light/dark cycles with free access to standard laboratory rat diet and water. The investigation conforms with the Guide for the Care and Use of Laboratory Animals published by the US National Institutes of Health (NIH) (publication no. 85-23, revised in 1996) and German animal protection code. All procedures with and handling of the animals during the investigations were reviewed and approved by the appropriate institutional review board (G-122/11).

### 2.2. Experimental Induction of Myocardial Infarction

The rats were anesthetized with a mixture of ketamine (100 mg/kg) and xylazine (3 mg/kg) intraperitoneally. An intratracheal tube was inserted, and the animals were artificially ventilated using a rodent ventilator (Föhr Medical Instruments GmbH, Seeheim, Ober-Beerbach, Germany). The core temperature (measured via a rectal probe) was maintained at 37°C with a controlled heating pad. The chest was opened via a left thoracotomy, followed by a pericardiotomy. A 6-0 single silk suture was passed around the left anterior descending (LAD) coronary artery, and the ends of the tie were pulled through a small pledget to form a snare and then tightened. After 45 minutes of ischemia, reperfusion was achieved by releasing the snare. After surgery the thorax was closed, the skin was sutured, and the rats were allowed to recover on a heating pad. Sham-operated animals were subjected to the same surgical procedures, except that the suture under the LAD coronary artery was not tied.

### 2.3. Experimental Groups

The rats were randomly divided into four groups: (1) W + sham: sham-operated nondiabetic Wistar control rats (*n* = 10); (2) GK + sham: sham-operated diabetic GK animals (*n* = 7); (3) W + MI: myocardial infarcted nondiabetic Wistar control rats (*n* = 9); (4) GK + MI: myocardial infarcted diabetic GK animals (*n* = 8).

### 2.4. *In Vivo* Hemodynamic Measurements and Cardiac Function

Twenty-four hours after the reperfusion, the animals were tracheotomised, intubated, and artificially ventilated. A polyethylene catheter was inserted into the left external jugular vein for fluid administration. A 2F microtip pressure-volume catheter was inserted into the right carotid artery and advanced into the ascending aorta. After being stabilized for 5 minutes, the arterial blood pressure was recorded and the catheter was advanced into the left-ventricle under pressure control. With the use of a special pressure-volume analysis program (PVAN, Millar Instruments, Houston, TX, USA), heart rate, systolic blood pressure, diastolic blood pressure, mean arterial pressure, ejection fraction, maximal slope of the systolic pressure increment (*dP*/*dt*_max_), and maximal slope of the diastolic pressure decrement (*dP*/*dt*_min_) were computed and calculated. The ventricular relaxation was assessed by the time constant of left-ventricular pressure decay (Tau). It was calculated by the Glantz method (Tau-g; regression of *dP*/*dt* versus pressure). The left-ventricular pressure-volume relations are assessed by transiently compressing the inferior vena cava. The slope *E*_max_ of the left-ventricular end-systolic pressure-volume relationship (ESPVR) was calculated as a load-independent index of LV contractility. At the end of each experiment, 0.1 mL of hypertonic saline (5%) was injected using the central venous line, and using the shift of pressure-volume relations, the parallel conductance volume was calculated by the software and used for the correction of the cardiac mass volume.

### 2.5. Biochemical Estimation

After 24 hours reperfusion had been established, the heart function was measured, and blood was collected from the abdominal aorta in Lithium-Heparin-Gel Monovette, EDTA, and serum tubes. After centrifugation, the plasma and serum samples were obtained. Plasma glucose levels, triglycerides, cholesterol, serum insulin levels, and urinary glucose levels were determined by the usage of an automatic biochemistry analyzer.

### 2.6. Histopathological Process

After the blood samples were collected, pieces of the left-ventricular myocardial tissue were collected for histopathology, fixed in buffered paraformaldehyde solution (4%), and embedded in paraffin. Then, 5 *μ*m thick sections were placed on adhesive slides and stained with hematoxylin and eosin (H&E). Cardiomyocyte cross-sectional areas were calculated under a microscope using the Cell^∧^A software (Olympus Soft Imaging Solutions GmbH, Germany). Masson's trichrome staining was performed to determine the extent of fibrosis. Four sections per heart were inspected under light microscopy and rated according to the following scoring system: grade 0 indicates normal tissue showing no fibrotic region, grade 1 indicates mild fibrosis, grade 2 indicates moderate fibrosis, and in grade 3 severe fibrosis is indicated. The histological evaluation was conducted by an investigator unaware of the treatment attribution of the animals.

### 2.7. Gene Expression Analysis Using RT^2^ Profiler PCR Arrays

After the blood samples were collected, pieces of the left-ventricular myocardial tissue were rapidly excised, frozen in liquid nitrogen, and stored at −80°C. The total RNA was extracted from the hearts with miRNeasy Mini Kit (Qiagen, Hilden, Germany) and was reverse-transcribed into cDNA using the RT^2^ First Strand Kit, mixed with RT^2^ qPCR Master Mix containing SYBR Green according to manufacturer's instructions (Qiagen, Hilden, Germany). The data was analyzed with the RT^2^ Profiler PCR Array Data Analysis Template available on the manufacturer's website.

### 2.8. Determination of Area at Risk and Infarct Size

After 24 hours reperfusion had been established, the heart function was measured, and blood samples were collected. After the heart was excised, a second investigator, unware of the randomization, determined the infarct size. The hearts were excised, quickly hung on a Langendorff apparatus, and perfused with 0.9% NaCl solution in order to wash the blood out from the coronary vessels. Then, the coronary artery was religated with the help of the 6-0 single silk suture previously located under the LAD coronary artery. Some additional rats in each experimental group (*n* = 3/group) were injected with 1.5 mL of Evans blue dye (1% w/v) via the aorta and coronary arteries to demarcate the ischemic risk (nonstained) or nonrisk (stained) area of the heart. The rest solely underwent 2,3,5-triphenyltetrazolium chloride (TTC) staining. The transverse heart tissue sections were incubated with 1% TTC for 30 minutes at 37°C. The viable myocardium was stained with brick red due to the formation of a precipitate that resulted from a reaction of TTC with dehydrogenase enzymes. The loss of these enzymes from the infarcted myocardium prevents the formation of the precipitate, and the infarcted area within the region at risk remains pale yellow. The tissue samples were then fixated in 4% formalin solution for at least 24 hours. The mean value of risk area has been calculated and used to determine the infarct size of the area at risk of infarction. Individual slices were photographed in colour using the Cell^∧^A software (Olympus Soft Imaging Solutions GmbH, Germany), and the extent of myocardial necrosis and the area at risk were quantified.

### 2.9. Chemical Reagents

Sodium pentobarbital was purchased from Merial GmbH (Hallbergmoos, Germany), and Evans blue and TTC were bought from Sigma-Aldrich (Steinheim, Germany).

### 2.10. Statistical Analysis

All data is expressed as mean ± standard error of the mean (SEM). In case of the PCR array gene expression, the *P* values were calculated based on Student's *t*-test of the replicate 2^−ΔCt^ values for each gene in the experimental groups. Genes corresponding to a *P* value less than 0.05 are indicated in Table 4. In all other cases, intergroup comparisons were performed by using a one-way analysis of the variance, followed by Student's unpaired *t*-test with Bonferroni's correction for multiple comparisons. A value of *P* < 0.05 was considered statistically significant.

## 3. Results

### 3.1. Biochemical Parameters

The biochemical values are listed in Table 1. The plasma glucose levels were significantly higher in diabetic rats compared to nondiabetic animals (GK + sham: 12.8 ± 1.5 mM versus W + sham: 8.5 ± 0.4 mM, *P* < 0.05), indicating the manifestation of an overt diabetes. Additionally, diabetic rats with myocardial infarction had significantly higher glucose levels when compared with the GK + sham animals (GK + MI: 19.0 ± 2.3 mM versus GK + sham: 12.8 ± 1.5 mM, *P* < 0.05). High levels of urinary glucose have been observed in diabetic rats compared to the nondiabetic animals (Table 1). We found no difference in the plasma cholesterol, triglyceride, and serum insulin concentrations amongst the experimental groups.

### 3.2. Myocardial Infarct Size

In rats, subjected to coronary artery occlusion and reperfusion, there was no difference in the area at risk between the nondiabetic Wistar and diabetic GK rats, indicating that a comparable degree of ischemic area was induced (Wistar 47 ± 8% versus GK 42 ± 2%, *P* = 0.42). However, the myocardial infarct size (infarct zone/area at risk) was smaller in diabetic hearts than in nondiabetic hearts (GK + MI: 27 ± 6% versus W + MI: 53 ± 9%, *P* < 0.05).

### 3.3. Myocardial Hypertrophy and Fibrotic Remodelling

**Figure 1:**
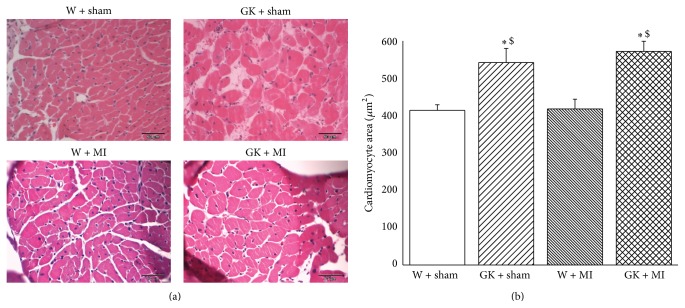
Histological analysis (hematoxylin and eosin staining). (a) Hematoxylin and eosin staining micrographs of transverse sections of myocardium (magnification ×400, scale bar: 50 *μ*m) and (b) quantitative analysis of cardiomyocyte cross-sectional area using measurements of ~20 cardiomyocytes in each group. Data is presented as mean ± SEM. ^*∗*^ *P* < 0.05 versus W; GK; ^$^ *P* < 0.05 versus W + MI. MI indicates myocardial infarction; GK: Goto-Kakizaki; W: Wistar.

**Figure 2:**
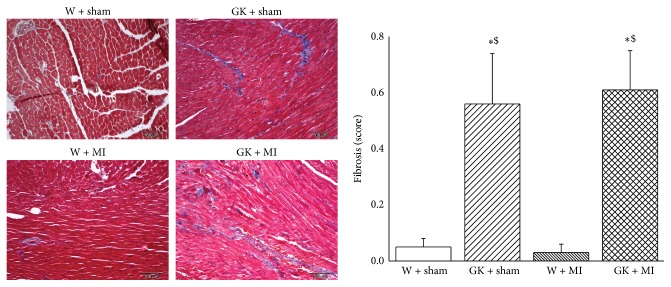
Histological analysis (Masson's trichrome staining). Histological examination of the myocardium stained with Masson's trichrome (magnification ×100, scale bar: 100 *μ*m). Data is presented as mean ± SEM. ^*∗*^ *P* < 0.05 versus W; ^#^ *P* < 0.05 versus GK; ^$^ *P* < 0.05 versus W + MI. MI indicates myocardial infarction; GK: Goto-Kakizaki; W: Wistar. Masson's trichrome staining (grade 0 indicates normal tissue showing no fibrotic region; grade 1 indicates mild fibrosis; grade 2 indicates moderate fibrosis, and grade 3 indicates severe fibrosis).

Compared to the corresponding age-matched control rats, the body weight was significantly lower, and the heart-to-body weight ratio was higher in diabetic animals (Table 2). Furthermore, the histological examination revealed that the cardiomyocyte transverse cross-sectional area in the GK + sham and GK + MI rats was significantly increased in the H&E-stained sections compared to the nondiabetic animals (Figure 1). Additionally, Masson's trichrome stained sections showed myocardial fibrosis in GK + sham and GK + MI rats compared to the nondiabetic animals (Figure 2).

### 3.4. Cardiac Pump Function

**Figure 3:**
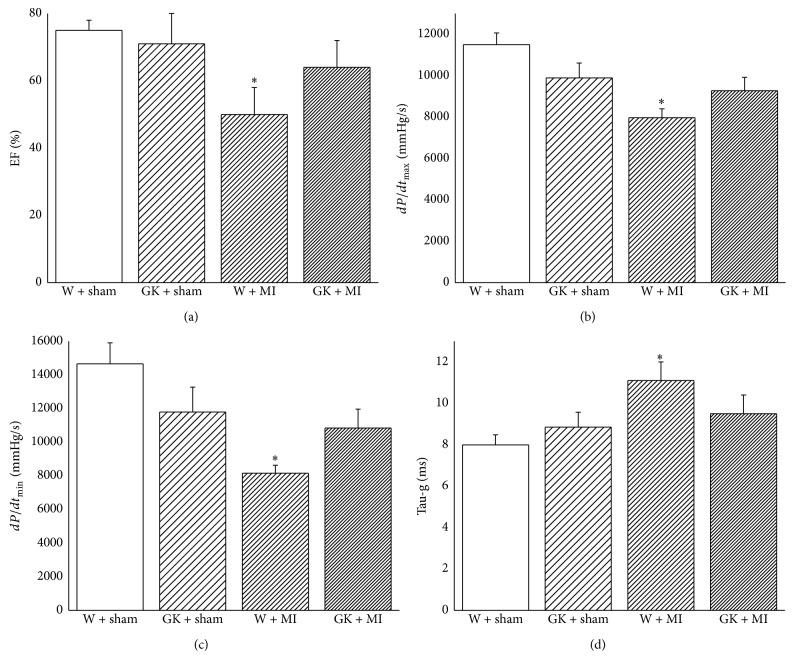
Left-ventricular systolic and diastolic function. Assessment of (a) ejection fraction, (b) maximal slope of the systolic pressure increment *dP*/*dt*_max_, (c) maximal slope of the diastolic pressure decrement *dP*/*dt*_min_, and (d) time constant of left-ventricular pressure decay Tau-g. Data is expressed as mean ± SEM. ^*∗*^ *P* < 0.05 versus W + sham. MI indicates myocardial infarction; GK: Goto-Kakizaki; W: Wistar.

The heart rate did not differ between the experimental groups (Table 2). The systolic blood pressure in GK + MI animals and the diastolic blood pressure and mean arterial pressure in GK + sham and GK + MI rats were significantly lower compared to the W + sham animals (Table 2). The cardiac index derived from a pressure-volume analysis is shown in Figure 3. Although the induction of acute myocardial infarction in nondiabetic rats significantly decreased systolic performance (ejection fraction, *dP*/*dt*_max_) and ventricular relaxation (*dP*/*dt*_min_, Tau-g), it had no effect on diabetic rats when compared to the corresponding control groups (Figure 3).

### 3.5. Myocardial Gene Expression Analysis Using RT^2^ Profiler PCR Arrays

**Figure 4:**
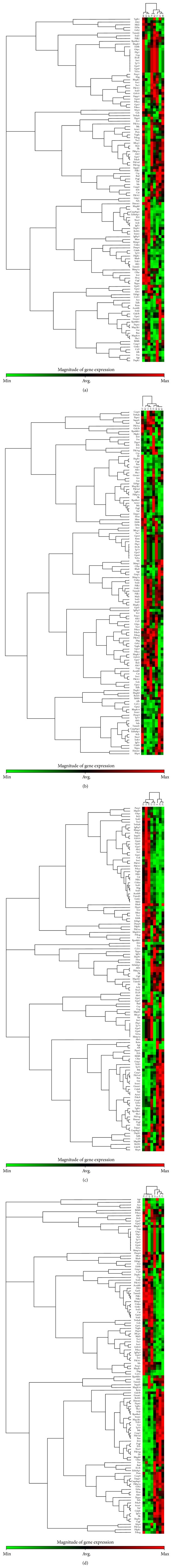
PCR array gene expression clustergrams: (a) GK + sham versus W + sham, (b) GK + MI versus GK + sham, (c) GK + MI versus W + MI, and (d) W + MI versus W + sham. MI indicates myocardial infarction; GK: Goto-Kakizaki; W: Wistar.

The significantly upregulated and downregulated 125 genes surveyed are shown in Tables 3 and 4 and in Figure 4. DM only significantly altered the expression of 5 genes (2 upregulated and 3 downregulated) in the hearts of GK + sham animals compared to W + sham rats. Only 4 genes (2 upregulated and 2 downregulated) showed significant expression changes in the hearts of GK + MI animals compared to GK + sham rats. However, 35 genes (19 upregulated and 16 downregulated) showed significant changes in expression in the hearts of GK + MI animals compared to W + MI rats. Genes whose expression was found to be highly up- and downregulated (fold change > +/−3) were mostly related to the onset, development, and progression of diabetes [glycerol-3-phosphate dehydrogenase (Gpd)-1, insulin-like growth factor binding protein (Igfbp)-5, phosphorylase glycogen liver (Pygl), heme oxygenase (Hmox)-1], apoptosis [cell death-inducing DFFA-like effector (Cidea)], antioxidants [superoxidase dismutase (Sod)-3, catalase], PI3K/Akt signaling pathway [pyruvate dehydrogenase kinase, isozyme (Pdk)-2, ribosomal protein S6 kinase polypeptide (Rps6ka)-l], cardiac fibrosis [matrix metallopeptidase (Mmp)-2, Gremlin-1], inflammation [tumor necrosis factor (Tnf)], and hypertrophy [myosin, light polypeptide (Myl)-2]. Acute myocardial infarction in nondiabetic rats (W + MI) altered 50 genes (19 upregulated and 16 downregulated) in comparison to the W + sham rats. These genes (fold change > +/−3) were mostly related to the PI3K/Akt signaling pathway [Rps6ka-l, bruton agammaglobulinemia tyrosine kinase (btk), Pdk-1, Pdk-2], inflammation [interleukin (Il)1b, Il6, Tnf], cardiac fibrosis [transforming growth factor (Tgf)-beta, Mmp2], hypertrophy [natriuretic peptide precursor A (Nppa), alpha actin (Acta)-1, FBJ osteosarcoma oncogene (Fos), Myl-2], ischemia/reperfusion injury [Hmox1], apoptosis [Cidea], and antioxidants [glutathione peroxydase (Gpx)-4, glutathione S-transferase kappa (Gstk)-1, thioredoxin reductase (Txnrd)2, sod3, catalase]. Additionally, the mRNA expressions of 4 genes corresponding to sod3, Il6, Il1b, and ctgf were highly regulated in W + MI compared to W + sham hearts, showing more than 11-fold change (fold changes for sod3: −14.7, Il6: +13.6, Il1b: +12.9, and ctgf: +12.0).

## 4. Discussion

The aim of this study was to investigate additional molecular pathomechanisms of acute myocardial infarction in mildly diabetic GK rats. In line with the previous literature [8, 12, 13], our results showed that DM decreases myocardial susceptibility to ischemia/reperfusion injury. We demonstrated that this short-term and mild diabetes-induced cardioprotection may be, at least in part, due to an increased transcriptional expression of markers of antioxidant defense and the prosurvival PI3K/Akt pathway and due to the downregulation of apoptotic genes, proinflammatory cytokine tumor necrosis factor-*α*, profibrogenic transforming growth factor-*β*, and hypertrophic marker alpha actin-1. This present work additionally expands previous studies by identifying additional target genes associated with the cardioprotective effect of middle diabetes against ischemia/reperfusion injury.

Diabetic cardiomyopathy, an early complication of diabetes, is a result of complex interactions between metabolic abnormalities (hyperinsulinemia, hyperlipidemia, and hyperglycemia) that accompany diabetes and their cellular consequences (oxidative stress, endothelial dysfunction, inflammation, and renin angiotensin aldosterone system activation), resulting in functional (systolic and diastolic dysfunction) and structural (left-ventricular hypertrophy and cardiac fibrosis) changes in the myocardium. In the present study, higher plasma and urine glucose levels, cardiomyocyte hypertrophy, and myocardial fibrosis were documented and the cardiac function was indistinguishable in GK rats when compared with nondiabetic animals. Despite these impairments, DM significantly altered only 5 of the tested genes involved in cardiac fibrosis (ctgf), hypertrophy (Nppa), and inflammation (Tnf, Il1b, Crp).

Although clinical trials showed that DM increased the sensitivity of the myocardium to ischemia/reperfusion injury [6, 7], infarct size studies in diabetic animal models have produced variable results. Some studies have shown that the diabetic heart is more sensitive to ischemic injury [14–16]; others found no difference [17, 18]. It has been also demonstrated that the myocardium of animals with DM compared to nondiabetic animals paradoxically is more resistant to ischemia, resulting in smaller myocardial infarctions [8, 12]. The reasons for the disparity between the animal and clinical data were the subject of a review in 1997, by Paulson, who concluded that the sensitivity of the diabetic heart to acute myocardial ischemia/reperfusion injury was dependent on the animal models and conditions used [9]. Additionally, in 2012, Whittington et al. added another complicating factor, the choice of the ischemia/reperfusion injury model, and the lack of other comorbidities such as age, dyslipidemia, and hypertension [19]. The duration and severity of the diabetic state, ischemic insults, and metabolic profiles after the diabetic induction play a role in explaining the vulnerability of diabetic hearts to myocardial ischemia/reperfusion injury. One of the reasons for the increased myocardial ischemic tolerance in diabetic animals may be associated with hypotension and bradycardia [20]. At the same time, different authors supported the endogenous cardioprotective phenotype (metabolic preconditioning) of the myocardium in the absence of differences in the hemodynamic measurements between control and diabetic animals [12]. In the present work, to evaluate the cardiac function, different indices were calculated. Our results showed that even though acute myocardial infarction decreased *dP*/*dt*_max_ (an index of contractility) and ejection fraction (widely used clinical parameter of systolic performance) in nondiabetic rats, it had no effects on diabetic animals. Additionally, the left-ventricular diastolic function, evaluated by *dP*/*dt*_min_ and Tau, has shown to be impaired by acute myocardial infarction in nondiabetic rats, while it was not significantly impaired in diabetic animals. However, we documented a modest hypotension in diabetic rats. Altogether, our results demonstrated that in the absence of differences in the cardiac systolic and diastolic functions, the infarct size was smaller in diabetic animals than in nondiabetic rats. However, the induction of a myocardial infarction in diabetic animals increased the glucose levels further when compared to the sham-operated diabetic animals. Furthermore, in the ischemic/reperfused diabetic myocardium only 2 of the tested genes were upregulated and 2 downregulated, when compared to diabetic hearts. This data suggests that an acute myocardial infarction in mild diabetic animals did not induce additional cardiac damage when compared to the noninfarcted diabetic rats.

Although the persistence of a hyperglycaemic environment prior to the ischemia/reperfusion injury may induce a myocardial adaptation [13], the exact mechanisms need to be elucidated. The following mechanisms have been proposed to explain the lower sensitivity of the acutely diabetic hearts to ischemia/reperfusion injury: the degree of intracellular acidity and how the cells handle this acidosis [21]. It has also been shown that preischemic myocardial glycogen content is increased in diabetic hearts [22, 23], and high glycogen reserves protect from ischemic damage resulting in smaller infarcts in the diabetic rat hearts [22]. Short-term diabetes may also increase the myocardial content of free-radical scavenging enzymes, catalase, and glutathione reductase which may also be involved [24, 25]. In the present study, we evaluated the effects of mild hyperglycemia after acute myocardial infarction on the ventricular transcriptional profile, using quantitative gene approaches. In line with these observations, our RT^2^ Profiler PCR Array results confirmed that reductions in the myocardial infarct area may be, at least in part, due to the upregulation of antioxidant enzymes including glutathione peroxidase-3 and peroxidase-4, glutathione S-transferase kappa-1, thioredoxin reductase-2, superoxide dismutase-3, and catalase.

Insulin sequentially activates the insulin receptor, PI3K, and Akt [26] through phosphorylation at threonine 308 and serine 473 [27]. Insulin receptor substrates- (Irs-) 1 and Irs-2 have been shown to play major roles in the control of cardiac homeostasis, metabolism, and function. Suppressing cardiac IRS-1 and IRS-2 may serve as a fundamental mechanism for induction of heart failure [28]. A growing body of evidence indicates that the PI3K/Akt pathway is generally considered to be beneficial for heart function [29]. The abnormal regulation of the PI3K/Akt pathway may be one of several factors contributing to diabetes-induced cardiac dysfunction, and its activation may prevent cardiac myocyte apoptosis and protect the myocardium from ischemia/reperfusion injury* in vivo* [30]. In the present work, we studied the prosurvival PI3K/Akt signal transduction pathway. An increased mRNA expression of Irs-1, Prkcz (a downstream of IRS-1 and PI3K), pyruvate dehydrogenase kinase (Pdk)-1, and Pdk-2 was observed in diabetic rats after myocardial infarction. Elevated inflammatory mediators have been shown to reduce the phosphorylation of tyrosine in IRS-1 and to reduce the activation of the PI3K/Akt pathway [31]. In line with these observations, genes encoding proteins, including markers of proinflammatory cytokine tumor necrosis factor-*α*, were downregulated in the ischemic/reperfused diabetic myocardium.

A programmed cell death mechanism or apoptosis plays a major role in the pathogenesis of diabetic cardiomyopathy. We showed that genes encoding proteins, including markers of apoptosis (cidea, Tp53, Bax, and caspase 3), were downregulated in diabetic hearts after acute myocardial infarction. Cardiac cell death can induce a compensatory hypertrophy of myocardial cells and reparative fibrosis [32]. Our results also showed that markers of myocardial fibrosis (Mmp-2, ctgf, and Grem-1), cardiac hypertrophy (myl-2 and acta1), and profibrogenic transforming growth factor-*β* were regulated in diabetic hearts after myocardial infarction. These findings provide new insight into the identification of potential genes that may be involved in mild hyperglycaemia environment induced protection against myocardial ischemia/reperfusion injury.

In the present study, our results showed that nondiabetic hearts are more sensitive to ischemia/reperfusion injury than the diabetic myocardium. Acute myocardial infarction impaired systolic performance (as evidenced by decreased *dP*/*dt*_max_ and ejection fraction) and ventricular relaxation (as shown by decreased *dP*/*dt*_min_ and prolonged Tau). The restoration of blood flow to the nondiabetic ischemic myocardium can cause injury. Ischemic damage and hypoxia induce the secretion of proinflammatory mediators, such as reactive oxygen species (ROS), cytokines, and chemokines, and cause ATP depletion. Furthermore, during reperfusion, the acute ischemic myocardium is subjected to several abrupt biochemical and metabolic changes including intracellular calcium overload, energy depletion, acidosis, activation of granulocytes, and the generation of reactive oxygen species [33]. During the postmyocardial infarction period, we showed that nondiabetic hearts responded with altered levels for transcripts representing markers of inflammation (upregulation of Il10, Tnf, Il1b, and Il6 expressions), apoptosis (upregulation of Tp53, Bax, and caspase 3 expressions), antioxidant defense (downregulation of Gpx3, Gpx4, Gstk1, Txnrd2, Sod3, and catalase expressions), and the prosurvival PI3K/Akt pathway (upregulation of Rps6k, Btk, Pik3cg, and Prkcb and down regulation of pdk2 and Prkcz expressions). These genes were inversely regulated in ischemic/reperfused diabetic hearts when compared to the nondiabetic myocardium. Hence, it is plausible to speculate that these genes may play an important role in the short-term induced cardioprotection against myocardial ischemia/reperfusion injury.

## 5. Conclusions

Our data shows that a mild hyperglycaemic environment provides protection to the heart against ischemia/reperfusion injury, at least in the early phase of the disease. Lists of genes demonstrating changes in their expression patterns are strongly influenced by the duration and severity of the diabetic state and ischemic insults. In our setup (45 min ischemic time and 24 h reperfusion), the downregulation of apoptotic genes, myocardial proinflammatory cytokine tumor necrosis factor-*α*, hypertrophic marker alpha actin-1, and profibrogenic transforming growth factor-*β* may at least be due to the activation of the prosurvival PI3K/Akt pathway and the upregulation of antioxidants during the acute phase of diabetes. From a clinical perspective, gene expression profiling studies aiming towards the discovery of pathways and identification of factors leading to coronary heart disease in diabetic patients, a high risk population, may help to design new preventive/therapeutic strategies.

A limitation of our study was that we did not investigate the protein expression of the genes in PRC array that were regulated but confined the investigation solely to profiling the myocardial transcriptome in diabetic rats after acute myocardial infarction.

## Figures and Tables

**Table 1:** Biochemical parameters in Goto-Kakizaki (GK) and control rats 24 h after sham operation or myocardial infarction.

	W + sham	GK + sham	W + MI	GK + MI
Plasma glucose [mmol/L]	8.5 ± 0.4	12.8 ± 1.5^*∗*^	8.4 ± 0.6	19.2 ± 2.3^*∗*#$^
Plasma triglycerides [mg/dL]	67 ± 9	60 ± 9	67 ± 7	88 ± 8
Plasma cholesterol [mg/dL]	70 ± 8	93 ± 6	71 ± 7	91 ± 7
Serum insulin [mU/L]	6.3 ± 0.8	5.3 ± 0.2	5.4 ± 0.5	5.1 ± 0.6
Urine glucose [g/L]	0.13 ± 0.02	0.73 ± 0.28^*∗*$^	0.14 ± 0.03	0.76 ± 0.15^*∗*$^

Data is expressed as mean ± SEM; ^*∗*^ *P* < 0.05 versus W + sham; ^#^ *P* < 0.05 versus GK + sham; ^$^ *P* < 0.05 versus W + MI; MI indicates myocardial infarction; GK: Goto-Kakizaki; W: Wistar.

**Table 2:** Physiological characteristics of Goto-Kakizaki (GK) and control rats 24 h after sham operation or myocardial infarction.

	W + sham	GK + sham	W + MI	GK + MI
Body weight [g]	450 ± 6	331 ± 8^*∗*$^	444 ± 11	322 ± 6^*∗*$^
Heart weight to body weight ratio [×1,000]	2.96 ± 0.08	3.73 ± 0.10^*∗*^	3.54 ± 0.10^*∗*^	4.05 ± 0.09^*∗*$^
Heart rate [beats/min]	453 ± 13	454 ± 28	456 ± 6	453 ± 4
SBP [mmHg]	145 ± 6	123 ± 6	137 ± 8	120 ± 7^*∗*^
DBP [mmHg]	117 ± 5	89 ± 6^*∗*^	113 ± 8	92 ± 8^*∗*^
MAP [mmHg]	126 ± 5	100 ± 6^*∗*^	121 ± 8	102 ± 7^*∗*^

Data is expressed as mean ± SEM; ^*∗*^ *P* < 0.05 versus W + sham; ^$^ *P* < 0.05 versus W + MI; MI indicates myocardial infarction; GK: Goto-Kakizaki; W: Wistar; SBP: systolic blood pressure; DBP: diastolic blood pressure; MAP: mean arterial pressure.

**Table 3:** Genes Table.

Official full name	Gene symbol	Official full name	Gene symbol
Acyl-coenzyme A dehydrogenase, short/branched chain	Acadsb	Interleukin 10	Il10
ATP citrate lyase	Acly	Interleukin 1 beta	Il1b
Actin, alpha 1, skeletal muscle	Acta1	Interleukin 6	Il6
Bcl2-associated X protein	Bax	Insulin receptor substrate 1	Irs1
Bruton agammaglobulinemia tyrosine kinase	Btk	Insulin receptor substrate 2	Irs2
Caspase 3	Casp3	Matrix metallopeptidase 2	Mmp2
Catalase	Cat	Myosin, light polypeptide 2, regulatory, cardiac, slow	Myl2
Chemokine (C-C motif) ligand 11	Ccl11	Natriuretic peptide precursor A	Nppa
Cell death-inducing DFFA-like effector a	Cidea	Pyruvate dehydrogenase kinase, isozyme 1	Pdk1
Cell death-inducing DFFA-like effector b	Cideb	Pyruvate dehydrogenase kinase, isozyme 2	Pdk2
C-reactive protein, pentraxin-related	Crp	Phosphoinositide-3-kinase, catalytic, delta polypeptide	Pik3cd
Connective tissue growth factor	Ctgf	Phosphoinositide-3-kinase, catalytic, gamma polypeptide	Pik3cg
Dipeptidylpeptidase 4	Dpp4	Phosphoinositide-3-kinase, regulatory subunit 1 (alpha)	Pik3r1
Ectonucleotide pyrophosphatase/phosphodiesterase 1	Enpp1	Protein kinase C, beta	Prkcb
FBJ osteosarcoma oncogene	Fos	Protein kinase C, zeta	Prkcz
Glucokinase	Gck	Protein tyrosine phosphatase, nonreceptor type 1	Ptpn1
Glycerol-3-phosphate dehydrogenase 1 (soluble)	Gpd1	Phosphorylase, glycogen, liver	Pygl
Glutathione peroxidase 3	Gpx3	Ribosomal protein S6 kinase polypeptide 1	Rps6ka1
Glutathione peroxidase 4	Gpx4	Superoxide dismutase 1, soluble	Sod1
Growth factor receptor bound protein 10	Grb10	Superoxide dismutase 2, mitochondrial	Sod2
Gremlin 1, cysteine knot superfamily, homolog (xenopus laevis)	Grem1	Superoxide dismutase 3, extracellular	Sod3
Glutathione reductase	Gsr	Sulfiredoxin 1 homolog (S. cerevisiae)	Srxn1
Glutathione S-transferase kappa 1	Gstk1	Transforming growth factor, beta 1	Tgfb1
Glutathione S-transferase pi 1	Gstp1	Tumor necrosis factor (TNF superfamily, member 2)	Tnf
Heme oxygenase (decycling) 1	Hmox1	Tumor protein p53	Tp53
Heat shock protein 1	Hspb1	Tribbles homolog 3 (Drosophila)	Trib3
Interferon gamma	Ifng	Thioredoxin reductase 2	Txnrd2
Insulin-like growth factor binding protein 5	Igfbp5		

**Table 4:** Significant upregulated and downregulated genes in Goto-Kakizaki (GK) and control rats 24 h after sham operation or myocardial infarction. MI indicates myocardial infarction; W: Wistar.

Functional groups	Genes	GK + sham fold change versus W + sham	*P* value

Cardiac fibrosis	Ctgf	+3.707	0.001532
Hypertrophy	Nppa	+6.2756	0.016233
Inflammation	Il1b	−3.1518	0.011374
Inflammation	Tnf	−3.1183	0.016066
Inflammation	Crp	−2.0963	0.035534

Functional groups	Genes	GK + MI fold change versus GK + sham	*P* value

Diabetes	Tnf	+5.6225	0.035036
Diabetes	Pygl	+3.1994	0.020738
Oxidative stress	Gstp1	−2.0236	0.021563
Cardiac fibrosis	Ccl11	−2.9549	0.035774

Functional groups	Genes	GK + MI fold change versus W + MI	*P* value

Diabetes	Gpd1	+6.641	0.001417
Diabetes	Igfbp5	+5.8232	0.01159
Diabetes	Ifng	+2.9216	0.005641
Diabetes	Irs1	+2.7073	0.028661
Diabetes	Irs2	+2.5629	0.026471
Apoptosis	Cidea	+6.8588	0.020129
Antioxidant	Sod3	+8.4477	0.017228
Antioxidant	Cat	+3.6086	0.013688
Antioxidant	Gstk1	+2.9415	0.013792
Antioxidant	Gpx3	+2.5874	0.005335
Antioxidant	Gpx4	+2.2143	0.008235
Antioxidant	Txnrd2	+2.4704	0.015564
Oxidative stress	Acadsb	+2.4938	0.008752
PI3K-AKT	Pdk2	+4.0001	0.01737
PI3K-AKT	Irs1	+2.7073	0.028661
PI3K-AKT	Prkcz	+2.4657	0.012351
PI3K-AKT	Pdk1	+2.4067	0.039144
Cardiac fibrosis	Mmp2	+4.4052	0.005183
Hypertrophy	Myl2	+4.069	0.004222
Diabetes	Hmox1	−3.3902	0.004516
Diabetes	Pygl	−3.0173	0.034296
Diabetes	Enpp1	−2.4937	0.008265
Diabetes	Tgfb1	−2.4518	0.001187
Diabetes	Pik3cd	−2.3951	0.005374
Apoptosis	Tp53	−2.5259	0.011446
Apoptosis	Casp3	−2.427	0.012582
Apoptosis	Bax	−2.1017	0.00374
PIK-AKT	Rps6ka1	−3.4451	0.002956
PIK-AKT	Pik3cg	−2.9438	0.009444
PIK-AKT	Btk	−2.7497	0.035023
PIK-AKT	Prkcb	−2.1591	0.003529
Inflammation	Tnf	−4.427	0.003721
Cardiac fibrosis	Grem1	−6.0096	0.040211
Cardiac fibrosis	Ctgf	−2.8267	0.039161
Hypertrophy	Acta1	−2.485	0.001472

Functional groups	Genes	W + MI fold change versus W + sham	*P* value

Diabetes	Pygl	+6.0176	0.012007
Diabetes	Pik3cd	+3.6417	0.00072
Diabetes	Trib3	+3.4219	0.021015
Diabetes	Enpp1	+2.8888	0.000436
Diabetes	Acly	+2.1769	0.000508
Diabetes	Ptpn1	+2.1691	0.005677
Apoptosis	Casp3	+2.8879	0.002393
Apoptosis	Bax	+2.5914	0.001228
Apoptosis	Cideb	+2.3322	0.000832
Apoptosis	Tp53	+2.0869	0.007736
Oxidative stress	Gsr	+2.0059	0.013533
Antioxidants	Srxn1	+2.4303	0.002489
PIK-AKT	Btk	+5.0358	0.009847
PIK-AKT	Rps6ka1	+4.6552	0.000443
PIK-AKT	Pik3cg	+2.9829	0.00643
PIK-AKT	Hspb1	+2.2659	0.020669
PIK-AKT	Prkcb	+2.1022	0.005081
Inflammation	Il6	+13.5898	0.009511
Inflammation	Il1b	+12.9385	0.027718
Inflammation	Tnf	+8.1656	0.000652
Inflammation	Il10	+2.814	0.027362
Cardiac fibrosis	Ctgf	+11.9928	0.000743
Cardiac fibrosis	Tgfb1	+3.647	0.000073
Hypertrophy	Nppa	+5.1685	0.02716
Hypertrophy	Acta1	+3.5345	0.000146
Hypertrophy	Fos	+3.3651	0.043876
Ischemia/reperfusion injury	Hmox1	+4.1327	0.004281
Diabetes	Gpd1	−8.3354	0.000051
Diabetes	Irs1	−4.025	0.008703
Diabetes	Igfbp5	−3.7655	0.0052
Diabetes	Ifng	−3.2748	0.016763
Diabetes	Gck	−3.1419	0.046149
Diabetes	Dpp4	−2.4258	0.019147
Apoptosis	Cidea	−6.5517	0.000294
Antioxidant	Sod3	−14.7384	0.000054
Antioxidant	Gstk1	−4.9174	0.000258
Antioxidant	Txnrd2	−3.3769	0.000067
Antioxidant	Gpx4	−3.1555	0.000135
Antioxidant	Gpx3	−2.9402	0.005985
Antioxidant	Sod1	−2.1671	0.000249
Antioxidant	Sod2	−2.0326	0.002173
Oxidative stress	Acadsb	−4.092	0.002653
Antioxidant	Cat	−4.6839	0.000134
PI3K-AKT	Pdk2	−6.0865	0.000008
PI3K-AKT	Pdk1	−4.2831	0.000002
PI3K-AKT	Pik3r1	−2.8108	0.009969
PI3K-AKT	Grb10	−2.4946	0.000153
PI3K-AKT	Prkcz	−2.3207	0.039517
Cardiac fibrosis	Mmp2	−5.9891	0.000011
Hypertrophy	Myl2	−4.5332	0.000579
